# The effectiveness of Forensic Outpatient Systemic Therapy in the treatment of juvenile antisocial behavior: A study protocol of a Multiple Case Experimental Design

**DOI:** 10.1371/journal.pone.0298057

**Published:** 2024-04-18

**Authors:** S. Marjolein van Cappellen, Hanneke E. Creemers, Larissa Hoogsteder, Joan van Horn, Jessica J. Asscher

**Affiliations:** 1 Department of Clinical Child & Family Studies, Utrecht University, Utrecht, Netherlands; 2 Faculty of Social and Behavioral Sciences, University of Amsterdam, Amsterdam, Netherlands; 3 Outpatient Forensic Mental Health Care Center, De Waag, Utrecht; PLoS ONE, UNITED STATES

## Abstract

**Background:**

Juvenile antisocial behavior can have long-lasting and devastating effects for juveniles themselves, victims, and society. Evidence-based treatment is vital. Forensic Outpatient Systemic Therapy (Forensische Ambulante Systeem Therapie; FAST) is a promising treatment for juveniles showing severe antisocial behavior including aggression, (domestic) violence, and delinquent behavior. FAST has a flexible intensity and length, addresses individual and systemic risk and protective factors, and is responsive to the abilities of the client (system), intervention characteristics all considered crucial for effective treatment. The current study will investigate whether FAST is effective in reducing aggression of the juvenile, reaching client formulated subgoals, and improving family functioning. Processes of change will be examined, as well as mediation by reaching client formulated subgoals and improved family functioning.

**Methods:**

A Multiple Case Experimental Design (MCED) with an ABC design will be performed (A = baseline, B = intervention, and C = follow-up). Juveniles with primary aggression and/or anger problems (*N* = 15) and their caregiver(s) will be recruited. Data collection will consist of self-report questionnaires and case file analysis. Participants fill out frequent short self-report questionnaires (twice a week during phase A, every other week during phase B, and every week during phase C) and two main questionnaires at the start of the intervention and immediately after intervention end, thereby covering a period of 5 to 11 months. Both visual and statistical analyses will be performed.

**Discussion:**

This study will generate robust knowledge and inform clinical practice on the effectiveness, processes of change, and mediating mechanisms of FAST, aiming to improve the treatment of future families within youth forensic care.

**Trial registration:**

This trial was registered at ClinicalTrials.gov on 28/08/2023, protocol ID 60-63600-98-1138a.

## Introduction

Juvenile antisocial behavior, including aggression, (domestic) violence, and delinquent behavior such as threatening, assault, property crime, and substance and weapon offences [1], can have long-lasting and devastating effects for victims [2, 3] and perpetrators. Victims experience long-lasting damaging consequences such as feelings of unsafety [4] and are at increased risk of developing social emotional problems [5, 6]. Juveniles exhibiting antisocial behavior have a heightened risk of out of home placement, delinquency, and recidivism [7]. They are less likely to have stable living situations, work environments, and relationships [8], induce high societal costs, and negatively affect societal safety [9]. Considering the long-lasting personal and societal consequences of juvenile antisocial behavior [8–10], evidence-based treatment is vital for not only the juveniles and their systems but also for society. Although various intervention programs are available for this target group, these programs generally do not achieve substantial and long-lasting effects in the reduction of antisocial behavior and recidivism [11, 12].

Forensic Outpatient Systemic Therapy (in Dutch: Forensische Ambulante Systeem Therapie; FAST) is an outpatient systemic intervention for juveniles (aged 12–21 years) who show antisocial behavior and their families, developed to (1) reduce juvenile antisocial and/or delinquent behavior; (2) prevent out of home placement; and (3) prevent or decrease recidivism (risk) [13]. Based on Bronfenbrenner’s socio-ecological theory [14], FAST aims to reach intervention goals by targeting juvenile, family, and systemic factors associated with the development and continuation of antisocial behavior, using components that originate from system therapy, cognitive behavioral therapy, aggression regulation therapy, and non-violent resistance.

In addition, FAST aims to be very adherent to the Risk Needs Responsivity (RNR) principles [15]. The RNR model states that, to be effective, interventions should adhere to several principles, among which the RNR are most important: (1) the risk principle: the intensity of the treatment should be adjusted to the recidivism risk; (2) the needs principle: the treatment should target the dynamic individual criminogenic needs; and (3) the (specific) responsivity principle: the treatment needs to be responsive to the abilities of the client (system) and needs to apply interventions that are effective in the target group. FAST has a flexible intensity and length, addresses risk and protective factors within the broad social context of a client system, and is responsive to the abilities of the client (system). In addition, if needed, FAST can be combined with other interventions to address specific individual risk factors of the client (system).

Previous pre-posttest studies showed that FAST resulted in positive changes on the desired outcomes: FAST had a large effect in reducing general recidivism risk, a moderate effect in decreasing problems in the emotional/personal functioning of the juvenile, and a small to moderate effect in improving family functioning [16]. However, more robust studies are needed to be able to attribute these results to the program offered. In addition, processes of change and the theorized mediators, i.e., whether changes in dynamic risk and protective factors result in decreased antisocial behavior, have not yet been investigated.

### Intervention subgoals

Based on both the RNR model [15] and the socio-ecological model [14], FAST subgoals have been formulated based on the dynamic risk and protective factors at the level of the individual, family, and the broader system of the juvenile to reach intervention goals. At the individual level, FAST aims to target juvenile criminogenic risk factors related to psychological functioning such as cognitive distortions [17–19] and low executive functions such as cognitive flexibility, inhibition [20, 21] and coping skills [22, 23]. In addition, FAST aims to effectuate adequate daytime activities [18, 24]. At the family level, FAST aims to improve family functioning, by increasing caregiver-juvenile relationship quality [25, 26] and caregiver behavior [27], and by decreasing caregiver-juvenile conflict [28]. At the level of the broader system, FAST aims to promote social support [29, 30], reduce interaction with deviant peers [31, 32], and decrease truancy [33].

### Aims of the study

The current protocol paper describes a Multiple Case Experimental Design (MCED) study investigating the effectiveness of FAST. The aim of the MCED is to investigate the effectiveness of FAST in reaching its primary intervention goal, i.e., reducing aggression, in reaching client formulated subgoals, and in improving family functioning (i.e., reducing juvenile-caregiver conflict and increasing caregiver responsiveness). In addition, the aim is to investigate processes of change within FAST, i.e., the onset, variability, trend, slope, and sequence of change [34] of the intervention (sub)goals. Further, the aim is to investigate whether reaching client formulated subgoals and improving family functioning indeed function as mediators in the effectiveness of FAST, i.e., whether they contribute to decreases in aggression [13].

Client formulated subgoals measure to what extent the FAST subgoals clients want to achieve during intervention are achieved. In addition, for all families, the FAST subgoal of improving family functioning will be investigated, by examining juvenile-caregiver conflict and caregiver responsiveness [13]. Both constructs have been shown to be crucial in achieving change in family functioning, and proposedly result in a decrease of aggression. Patterson [35] proposed that caregiver-juvenile conflict involves coercive interaction cycles that lead to the development of juvenile conduct problems. Indeed, longitudinal studies have shown caregiver-juvenile conflict to predict juvenile antisocial behavior [36] and aggression [37]. In addition, multiple (meta-analytic) reviews have shown that lower caregiver responsiveness (i.e., feeling insufficiently equipped in parenting skills) is associated with increased externalizing behavior of juveniles [38, 39], especially for samples of older children [38].

The hypotheses are that (1) FAST is effective in reducing aggression; (2) FAST is effective in reaching client formulated subgoals; (3) FAST is effective in improving family functioning (i.e., decreasing juvenile-caregiver conflict and increasing caregiver responsiveness); and (4) improvements in client formulated subgoals and family functioning mediate FAST effectiveness.

In addition to (quasi) experimental studies, MCEDs are increasingly used to study intervention effectiveness in youth populations [40]. The current study is part of a larger research project to determine the effectiveness of FAST and compliments a randomized controlled trial (RCT) [41] because of several design-specific benefits. First, MCEDs can capture within-person changes and intervention effects [40] that might not be detected using group-level analyses [42, 43]. Individual variability in response to treatment seems overlooked in group-level studies on youth interventions [44], highlighting the importance of combining group-level studies with MCEDs. Second, MCEDs allow a precise study of processes of change [45]. The multiple assessment points of an MCED allow detailed documentation [46] to examine the onset, variability, trend, and slope of change and the sequence of changes across different constructs [34]. In addition, the frequent measurements allow monitoring of micro-processes, whilst increasing ecological validity by reducing recall bias [47, 48]. Thereby, MCEDs allow the identification of small behavioral or mental changes. Third, the multiple measurements within each phase of the MCED design allow for a relatively small sample size [45]. Fourth, the design allows to assess if the intervention effect and mediating mechanisms vary between participants, and therefore to what extent effects and mechanisms can be generalized to other cases [44]. Previous MCED intervention studies have found individual differences in the processes of change [49–51], substantiating the importance of utilizing this design in intervention research.

## Methods

### Design

**Fig 1:**
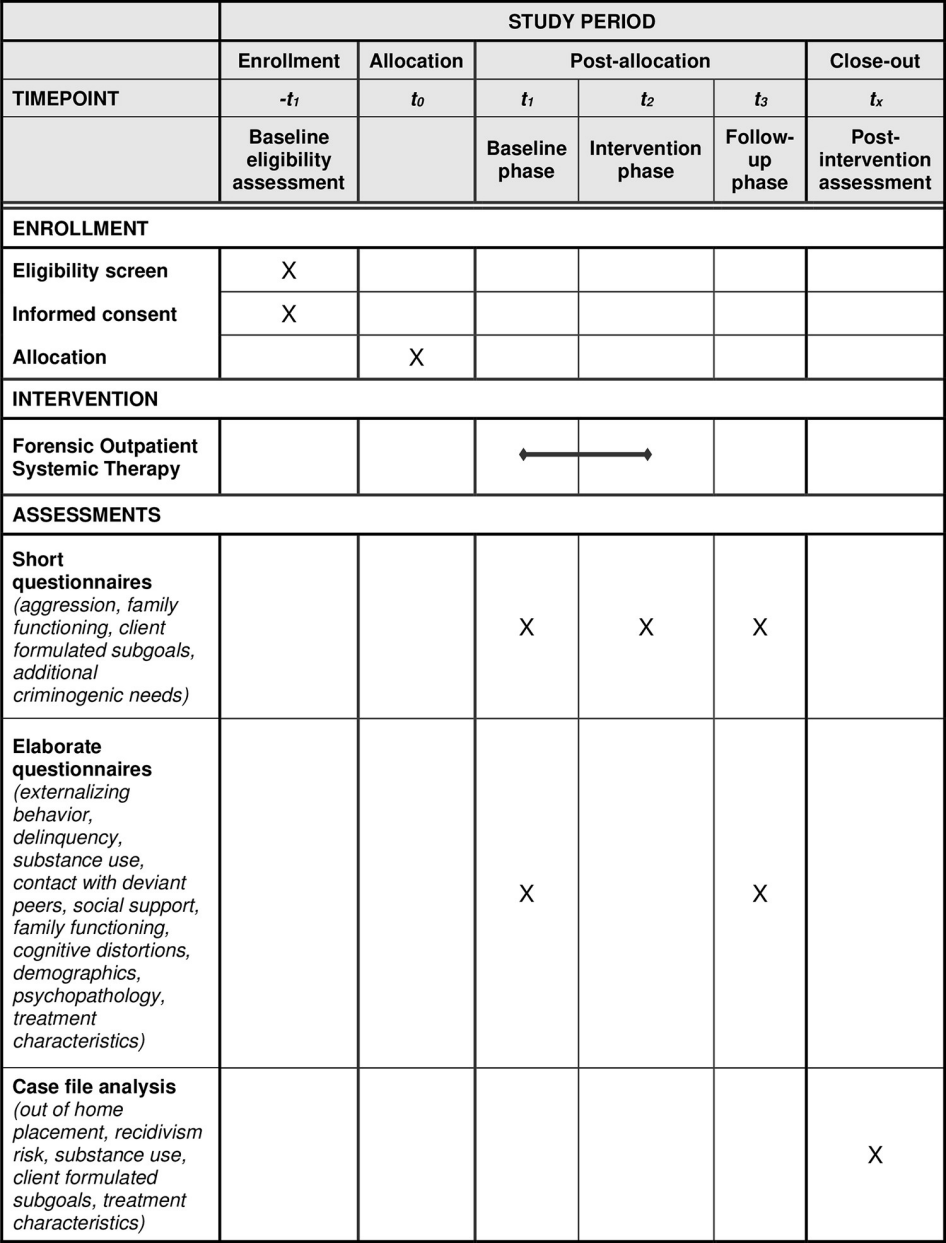
SPIRIT schedule of enrollment, interventions, and assessments.

**Fig 2:**
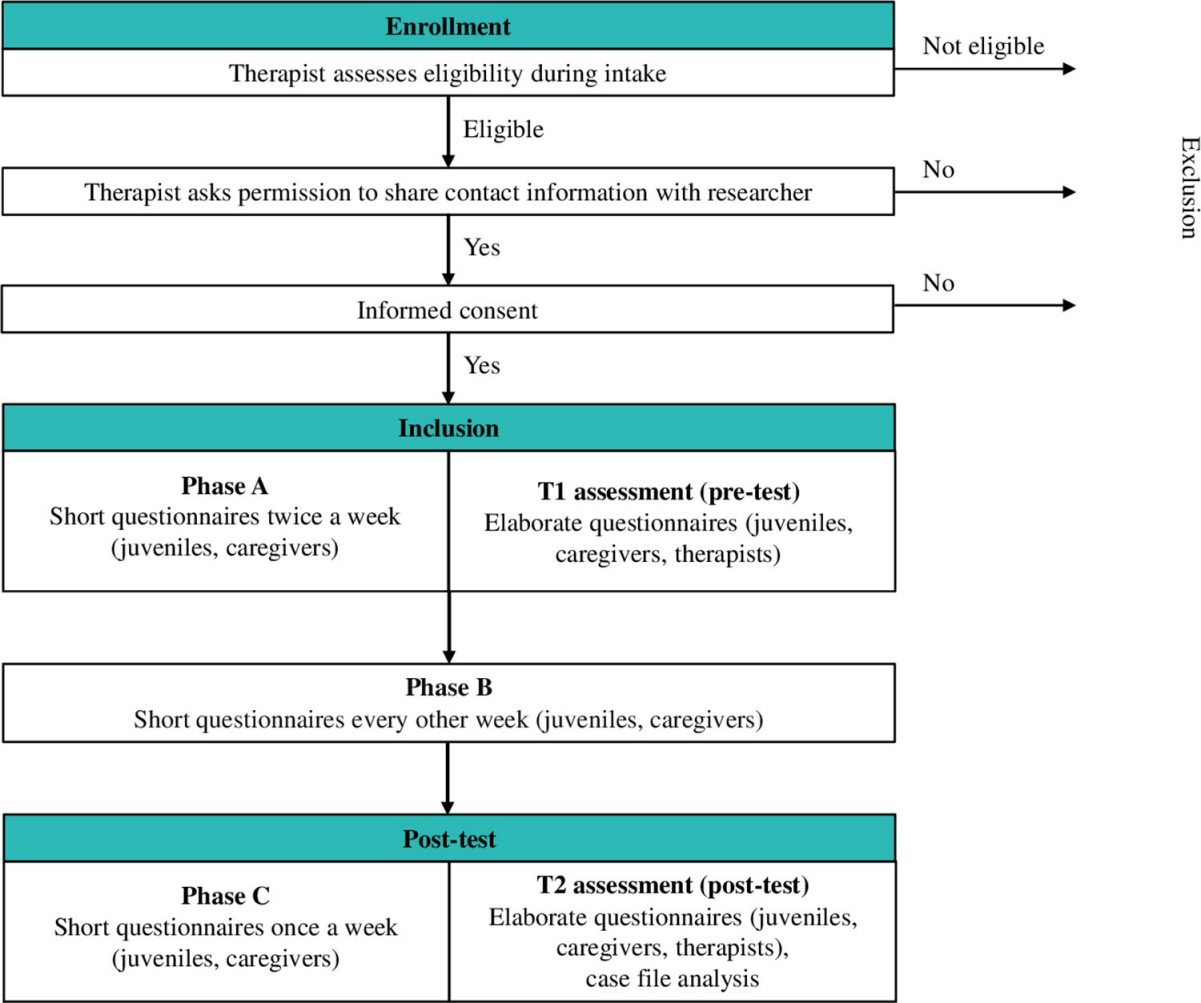
Conceptual model of study design.

An ABC design will be used with at least five assessment points per phase [45]. Phase A (baseline) covers the first 3–5 weeks of FAST during which the preconditions to enter treatment are created. For all families this part involves a focus on risk management, and for some families it involves a focus on exploratory diagnostics. Phase B covers the subsequent period of 2–8 months, starting with preliminary treatment that is replaced by customized treatment once the treatment plan has been finalized. Phase C (follow-up) covers six weeks after intervention end. The study has a multi-informant (juveniles and caregivers) design. Participants complete short questionnaires during phases A, B, and C with varying frequency: twice a week during phase A, every other week during phase B, and every week during phase C. Additionally, participants fill out two more elaborate questionnaires at the start of FAST and after finishing FAST, and case-file analysis is conducted. The study was registered at ClinicalTrials.gov on 28/08/2023 (protocol ID 60-63600-98-1138a). See Fig 1 for the SPIRIT schedule of enrollment, interventions, and assessments, Fig 2 for a conceptual model of the study design, and S1 Checklist for the SPIRIT checklist.

### Setting

FAST is offered by de Waag, an outpatient forensic mental health care center in the Netherlands with 12 treatment sites. Clients are referred by the juvenile justice system or voluntarily by mental healthcare professionals, school care coordinators, and general practitioners. FAST therapist teams at seven treatment locations will approach participants for the study. Participant recruitment started on September 26^th^ 2023. Participants will be recruited until August 2024. The expected complete date range for participant recruitment and follow-up is September 26^th^ 2023 to July 2025.

### Participants

It is currently unknown how many case series should be included in an MCED study to provide evidence of intervention effectiveness [52]. Numbers range from 3 [53] to 9 [54] or 10 case series [55]. Therefore, complete data will be collected for at least *N* = 10 families. Taking drop-out of treatment and/or the study into account, *N* = 15 juveniles and their caregiver(s) will be recruited. The target group of FAST is diverse in terms of (comorbid) problems, but approximately 93% of the referred juveniles has a behavioral disorder [13] and they often grow up in families with multiple and complex problems. In FAST, 75% of juveniles is male.

#### Inclusion and exclusion criteria

FAST therapists assess whether clients meet the inclusion and exclusion criteria of FAST during the standard intake procedure of FAST. The FAST inclusion criteria are: (1) Juvenile has an estimated IQ-score of 80 or higher and/or sufficient adaptive skills to benefit from FAST; (2) Juvenile is aged 12 to 21 years old at the start of the intervention; (3) Juvenile exhibits externalizing behavior resulting in problems in at least two life areas (family, school, or leisure time), determined by clinical impressions based on information from intake and/or referrer information; (4) Juvenile has a medium to high recidivism risk, measured by the Risk Assessment Instrument for Outpatient Forensic Mental Health Care Youth (RAF GGZ Youth) [56]; (5) Presence of juvenile-caregiver relationship problems, measured by the RAF GGZ Youth; (6) Juvenile has a diagnosis of a DSM-5 behavioral disorder, which is determined using a new diagnostic process or case file analysis; (7) Juvenile and caregiver(s) cannot be motivated to follow treatment at the treatment site after multiple attempts by the therapist; and (8) Juvenile resides with their caregiver(s) or is expected to return to residing with their caregiver(s) within the first two months of FAST. The FAST exclusion criteria are: (1) Juvenile exhibits severe psychiatric symptoms requiring admission; (2) Problem behavior of the juvenile is caused primarily by substance abuse problems, and it is expected that treatment of the substance abuse problems will decrease the problem behavior; and (3) The safety of the family members or therapist cannot be sufficiently guaranteed.

To be eligible for participation in this specific study, one modified study inclusion criterium applies, i.e., the juvenile has primary aggression and/or anger problems (approximately 80% of referred juveniles). Thereby, juveniles exhibiting primarily sexually transgressive behavior, truancy, or property crimes are excluded from the study. In addition, one study exclusion criterium applies, i.e., the juvenile is in secure residential care or confined in a correctional or detention facility at the start of the intervention.

### Procedure

During intake, therapists evaluate whether clients meet the eligibility criteria and ask juveniles and/or caregiver(s) permission to share their contact details with the researcher. If they agree, FAST clients will be approached by the main researcher or research assistants, whom all have signed a non-disclosure agreement and provided a certificate of conduct, and receive verbal and written information about the study. The researcher obtains written informed consent from juveniles and caregivers for own participation, and from caregiver(s)/legal representative(s) for juveniles younger than 16 years.

During the study, participants fill out short questionnaires that contain approximately 37 items, taking about six minutes [57]. In addition, participants (juveniles and caregivers) complete two more elaborate questionnaires (30 minutes): prior to, or during the first weeks of intervention (pre-test; T1) and immediately after intervention (post-test; T2). These data are used for more extensive assessment at the start and end of the intervention and to assess background information of participants. Therapists fill out two questionnaires (5 minutes) at T1 and T2, providing background information on therapist characteristics. Further, case file analysis will be used to retrieve information on the primary diagnosis of the juveniles, out of home placement, and questionnaires that are filled out as part of the standard FAST procedure by juveniles, caregivers, and therapists.

Given the complexity of the problems the target group faces, which often adversely affects their motivation to participate in treatment or research, the data collection is adjusted to the preferences and agenda of the participants for timing and location (e.g., by (video) phone calls or at the homes of the families). Trained research assistants are available to assist with filling out the questionnaires, e.g., by taking them in interview form. Participants receive financial compensation for filling out the questionnaires: 2.50 euros per short questionnaire and 15 euros per elaborate questionnaire. When completing all measurements, participants receive an average total of 95 euros.

### Intervention

The treatment stage of FAST lasts five to nine months depending on the individual goals of the juvenile and the caregiver(s) and is followed by a period of aftercare. For more information on the treatment stages of FAST, see van Cappellen et al. [41]. At the start of treatment, therapists write an individualized basic Empirical Intervention cycle Summary (EIS). In the basic EIS, a problem analysis or function analysis of the problem behavior is described. The recidivism risk is determined, and the safety of the juvenile, caregiver(s), and therapists and the degree of motivation are described. The basic EIS describes which FAST subgoals need to be targeted to realize the main goal of FAST, as agreed upon between therapist, juvenile, and caregiver(s). During treatment, therapists evaluate the EIS every two weeks with the juvenile and the caregiver(s) and discuss which general and optional FAST subgoals have the most priority. Interventions are selected based on the chosen subgoals and by applying analysis circles. An analysis circle is created around a problem that is related to the chosen FAST subgoal: On the right side of the circle, the influencers that contribute negatively to the problem behavior, or increase the problem behavior are described; on the left side of the circle, the influencers that reduce the problem behavior are described. Influencers can originate from various systems around the juvenile and family and are introduced by the juvenile and caregiver(s) themselves. When it is determined that the chosen subgoals are reached, new goals are prioritized and new analysis circles are made. During treatment, the following supplementary modules can be selected for individual treatment: Stress and anger reduction, Impulse control, Self-control, Perceiving and interpreting correctly, Emotion regulation, and Self-image. Every two months an evaluation takes place to determine whether longer treatment is needed, with a maximum of nine months. In the last stage of the treatment, a future plan is developed that aims to prevent relapse.

The level of program integrity, indicating whether treatment is delivered according to the method and treatment manuals, can affect treatment results [58, 59]. Within FAST, treatment integrity is monitored closely. Every FAST therapist has succeeded the FAST basic training and offers FAST minimally 20 hours per week. Each team has weekly FAST team meetings, during which treatments are monitored by evaluating the EIS’ and a bi-monthly treatment checklist, guided by an appointed supervisor who is responsible for treatment integrity. At the end of the treatment, the FAST evaluation forms are completed by juveniles, caregivers, and therapists to verify compliance with the most essential FAST methods and techniques. FAST includes around 3 hours of face-to-face direct treatment time weekly and consists of a maximum of 10% online direct treatment time (i.e., treatment via phone, video-calling, or texting).

### Measures

The data collection and measures used in the elaborate questionnaires and case-file analysis are described in detail in the study protocol of our RCT [41]. The short questionnaires contain approximately 37 items (see below). All items on the short questionnaires are answered on a Visual Analog Scale (VAS) ranging from 0 to 100, as this is a more sensitive way to measure change [57]. To reduce the likelihood of a learning effect, question order will be randomized for each measurement. An overview of the various constructs, instruments, and respondents of the short assessments is presented in Table 1.

**Table 1:** Concepts, instruments, and respondents of the short assessments.

Concept	Instrument	Respondent	
		Juvenile	Caregiver
Aggression	YSR	x	
	CBCL		x
Family functioning	NRI	x	x
	NPQ	x	x
Client formulated subgoals	FAST Goal list	x	x

YSR = Youth Self Report; CBCL = Child Behavior Checklist; NRI = Network of Relationship Inventory; NPQ = Nijmeegse Parenting Questionnaire.

### Aggression

Aggression will be measured using the Aggressive Behavior scales of the Youth Self Report (YSR) [60] and the Child Behavior Checklist (CBCL) [61]. Both scales consist of 19 items. Example items are “I lie or cheat” for the YSR and “Lying or cheating” for the CBCL.

### Client formulated subgoals

Client formulated subgoals will be measured using the FAST Goal list, which consists of 21 items asking respondents to rate their current functioning on the general primary and secondary goals of FAST. An example item is “I receive sufficient support from people close to me (acquaintances, friends, or family)”. The FAST Goal lists are used in an idiographic approach [62]. The first short questionnaire contains the entire FAST Goal list, and a follow-up question asks participants to select the top three FAST goals they will be working on during FAST as discussed with their therapist during intake. In the subsequent measurements, only the items measuring the three prioritized goals are administered. Added to these three items, a fourth item measures whether prioritization or goals have changed. If so, the participant is asked to state their new prioritization or goals, and the questions about the new goals are added to the subsequent measurements.

### Family functioning

Juvenile-caregiver conflict will be measured using the adolescent and parent versions of the Network of Relationship Inventory (NRI) [63], containing six items. An example item is “How much do you and your parents disagree or argue with each other?” for juveniles and “How much do you and your child disagree or argue with each other?” for caregivers. Caregiver responsiveness will be measured using the Nijmeegse Parenting Questionnaire (NPQ) [64], containing eight items. An example item is “If I’m sad or worried about something, my caregiver notices” for juveniles and “If my child is sad or worried about something, I notice it” for caregivers.

### Additional criminogenic needs

If severe truancy, substance use, contact with deviant peers, or delinquent behavior is reported at T1 but not prioritized in the top three goals, single items will be administered on these problems as well. For example, if the juvenile reports severe substance use in the T1 questionnaire, but reducing substance use is not identified as a goal in the prioritization of client formulated subgoals, an item of the Peilstation Middelengebruik [65] is added. An example item is “How many times have you used weed in the past two weeks?”

### Data management

Contact data are stored in a digital double encrypted database. Research data are stored and analyzed in separate files, without direct links to the participants. Participants fill in questionnaires on paper or online using personalized links from the online survey tool of Utrecht University (Qualtrics). All completed paper documents are stored secured at Utrecht University and will be scanned and directly stored on YODA, a research data management service that is compliant to the guidelines of the General Data Protocol Regulation. Completed paper questionnaires will additionally be entered into Qualtrics. Information from case file analysis will be coded into SPSS or JASP files. All data will be stored directly on YODA and only the researchers involved in this study have access to the data.

### Plan of data analysis

The methods of analyzing MCED data are developing rapidly [66]. The current best practice suggests combining visual analysis with statistical analyses [45, 67], but there is no consensus about which statistical analyses are best [66]. Based on currently available knowledge, we expect to perform visual analysis following the systematic protocol of Wolfe et al. [68] and statistical analyses using simulation modeling analysis (SMA) [69], as it is suitable with a relatively small number of observations per phase (i.e., N < 30) [70]. A bootstrapping method enables the analysis of variable changes across phases while accounting for autocorrelation. Pearson correlations will be calculated across phases per variable and per participant for level changes (i.e., changes in mean levels) and slope changes (i.e., changes in data patterns). In addition, cross-lagged correlations within phase will be investigated [70]. The Percentage of Non-overlapping Data will be provided for clinically relevant level changes (i.e., > 80%) [71] and a Bonferroni correction will be applied to adjust for multiple comparisons.

## Discussion

This study protocol describes an MCED investigating the effectiveness of FAST in reducing aggression of juveniles with antisocial behavior, achieving client formulated subgoals, and improving family functioning. In addition, change processes will be examined by investigating the onset, variability, trend, slope, and sequence of change of the intervention (sub)goals, and mediation by reaching client formulated subgoals and improved family functioning will be assessed. The hypotheses are that (1) FAST is effective in reducing aggression; (2) FAST is effective in reaching client formulated subgoals; (3) FAST is effective in improving family functioning (i.e., decreasing juvenile-caregiver conflict and increasing caregiver responsiveness); and (4) improvements in client formulated subgoals and family functioning mediate FAST effectiveness.

In the data collection of this study, we anticipate several challenges. One will be receiving informed consent from participants quickly to have sufficient measurements in the baseline phase (phase A). A second challenge will be the recruitment and retainment of *n* = 15 FAST clients over a period of 5 to 11 months, as the target group is generally hard to reach and motivate for research. During the data collection of our other studies on the effectiveness of FAST [41], families voice to be overloaded by the multiple problems they experience in their daily lives. Because FAST is (necessarily) an intensive treatment program, families can experience the additional burden of participating in scientific research as being too high. The frequent and prolonged measurements that are essential in the MCED design might therefore discourage participation. The current study thus requires special attention for the recruitment and retainment of the families. First, it will be key to adapt our data collection as much as possible to the needs and wants of the participants. As we do in our other studies [41], we will adjust the timing, the location, and the way of completing the measurements to their preferences. In addition, to contribute to the formation of a good working relationship, we will try to let the same researcher conduct all assessments with a participant [72]. We will compensate participants financially for each completed measurement, as this increases motivation to participate [73, 74]. Second, conducting scientific research with this target group requires a strong collaboration with clinical practice and therapists [72]. As we have already started conducting an RCT investigating the effectiveness of FAST [41], we have established a strong collaboration with the participating therapist teams. We will continue to put effort and time into our collaboration.

The proposed MCED study meets nearly all quality criteria in the existing guidelines [53], but a limitation of our study design is that the ABC design does not include systematically manipulated baselines across families, as the duration of phase A is determined by the therapist. This poses potential threats to the internal validity of our results [45]. However, as highlighted in the article of Kratochwill et al. [45], conducting an extended baseline is unethical when participants exhibit behavior that is dangerous to the participants themselves or others [75]. As the target group of the current study exhibits severe antisocial behavior which poses risk for themselves, their surroundings, and society and requires immediate treatment, extending the baseline phase can be seen as an unacceptable risk. We have designed this MCED study in a way that makes it safe to conduct in the reality of clinical practice and that increases external validity.

Despite this limitation and the potential challenges, the current study has several important strengths. First, the MCED design has several strengths (see Introduction). Most importantly, the design allows to study the within-person intervention effects [44] and possible individual variability in processes of change [45] during the course of FAST. Second, the study uses an idiosyncratic measure of client formulated subgoals. Thereby, it measures treatment effect on the problems clients have discussed with their therapist to be the most important. Additionally, this approach provides a complementary and more specific measurement of problems than the more broad and generic measurement in standardized assessment [62]. Third, the multi-informant (juveniles and caregivers) design of the study allows us to compare the reports of juveniles and caregivers on intervention effectiveness and course. This seems especially important in youth intervention research, as the points of view of juveniles and caregivers can differ considerably [62]. Fourth, after affirming that the sample of FAST clients in this MCED study is representative of the bigger sample of clients that participate in our RCT or quasi-experimental study, we will be able to infer whether the processes of change and mediating mechanisms of FAST that are found in this study also apply to the larger FAST target group.

This study will improve knowledge on potential within-person processes of change, including mediations, in a systemic intervention targeting juvenile antisocial behavior and their multi-problem families. Next to investigating whether and how improving family functioning contributes to a decrease in aggression [38, 39], this study will investigate if, how, and in what order client subgoals formulated at the beginning of the intervention are reached, and whether improvements contribute to decreases in aggression. Thereby, this study will allow to assess whether intervention customization, in the form of adjusting the intervention subgoals to the specific criminogenic factors present in the client system [13], indeed contributes to intervention effectiveness and what the underlying processes of change are. Further, the examination of change processes on an individual level can inform clinical practice on what course of change to expect during intervention. Following, clients can be informed and questions can be answered based on evidence: When will change likely occur? What process of change can be expected: Is change stable or variable? Is it worth investing in a specific client formulated subgoal first to reach the main goal, i.e., a decrease in aggression? This knowledge might help to motivate families to push through the difficult times of intervention, or to invest in specific intervention goals. In short, the results of this study will yield knowledge on the processes of change and mediators of FAST, potentially contributing to evidence-based decision making in clinical practice, informing families and therapists on what to expect during treatment.

## Conclusion

The present MCED study aims to investigate the effectiveness, change processes, and mediating mechanisms of FAST. Evidence-based treatment for juveniles with antisocial behavior is crucial for juveniles themselves, for their families, and for society. This MCED will allow us to generate robust knowledge to inform clinical practice on which processes of change, including mediators, contribute to the effectiveness of systemic interventions targeting juveniles with antisocial behavior on an individual level. Thereby, the results potentially improve the treatment of future families within youth forensic care.

## Supporting information

S1 Checklist SPIRIT 2013 checklist: Recommended items to address in a clinical trial protocol and related documents*. (PDF)

S2 Checklist TREND statement checklist. (PDF)
